# Epithelial cell adhesion molecule in human hepatocellular carcinoma cell lines: a target of chemoresistence

**DOI:** 10.1186/s12885-016-2252-y

**Published:** 2016-03-16

**Authors:** Yan Li, Russell W. Farmer, Yingbin Yang, Robert C. G. Martin

**Affiliations:** Division of Surgical Oncology, University of Louisville School of Medicine, Louisville, KY 40202 USA; School of Life Science, Southwest University, Chongqing, 400716 China; Department of Surgery, Division of Surgical Oncology, University of Louisville School of Medicine, 315 E. Broadway - #312, Louisville, KY 40202 USA

**Keywords:** Hepatocellular carcinoma, Epithelial cell adhesion molecule, Doxorubicin, 5-FU, Cisplatin

## Abstract

**Background:**

The low survival rate of hepatocellular carcinoma (HCC) is partly attributable to its resistance to existing chemotherapeutic agents. Until now, there have been limited chemotherapeutic agents for liver cancer. Epithelial cell adhesion molecule (EpCAM) has been found to be over-expressed during stages of carcinogenesis and has been associated with poor overall survival in many cancers. The aim of this study was to evaluate EpCAM expression in HCC and evaluate the effects of EpCAM to established chemotherapy.

**Methods:**

Three human hepatocellular carcinoma cell lines—HepG2, Hep3B and HuH-7—were pre- and post-treated with doxorubicin, 5-fluorouracil (5-FU) and cisplatin. Cell viability and EpCAM protein expression were measured by MTT assay and Western Blotting respectively. EpCAM positive cells were analyzed by flow cytometry. To evaluate the effects of doxorubicin efficacy on EpCAM positive cells, a small interfering RNA (siRNA) specific to EpCAM was transfected into the cells and treated with doxorubicin. Results: EpCAM was significantly down-regulated by doxorubicin treatment in all three HCC cell lines (*P* <0.05 or 0.01). EpCAM expression was down-regulated by the 5-FU and cisplatin in HepG2 cells, however the EpCAM expression was up-regulated by 5-FU and cisplatin in Hep3B cell line. EpCAM expression was down-regulated by 5-FU, and up-regulated by cisplatin in Huh-7 cell line. Flow cytometry assay showed doxorubicin exposure decreased EpCAM positive cell quantities in three HCC cell lines. EpCAM siRNA knock-down attenuated cell mortality after doxorubicin exposure.

**Conclusion:**

All of these findings demonstrate that EpCAM is one of targets of chemoresistence.

## Background

Hepatocellular carcinoma (HCC) is the third most common cause of cancer-related mortality worldwide [1]. It is the most common type of liver cancer. The 5-year survival rate of liver cancer patients in the United States is very low; it is the second most lethal cancer after pancreatic ductal adenocarcinoma [2]. The low survival rate of liver cancer partly comes from its resistance to existing chemotherapeutic agents [3]. At this time, there are no perfect anticancer chemotherapeutic agents for liver cancer.

5-fluorouracil (5-FU), cisplatin and doxorubicin are commonly used therapeutic agents in the clinical setting. The fluoropyrimidine 5-FU is an antimetabolite drug that is widely used for the treatment of cancer, particularly for colorectal cancer. It works through noncompetitive inhibition of thymidylate synthase and incorporation of its metabolites into RNA and DNA [4]. Inside the cell, 5-FU is transformed into different cytotoxic metabolites and induces cell cycle arrest and apoptosis by blocking the cell’s ability to synthesize DNA. Specifically, 5-FU interferes with the synthesis of deoxythymidylate (dTMP). Without dTMP, rapidly dividing cancerous cells were induced into thymineless death. In addition, 5-FU has been reported to inhibit the activity of the exosome complex, which is essential for cell rRNA processing [5].

Cisplatin is a platinum-based chemotherapy drug used to treat various types of cancers. Inside a cell, cisplatin forms a platinum complex that binds to and cross-links DNA. This cross-linking damages DNA and repair mechanisms are activated. Once the repair mechanisms damaged, the cells are found to not be salvageable, the death of those cells is triggered through apoptosis.

The exact mechanism of action of doxorubicin is complex and still somewhat unclear, though it is thought to interact with DNA by intercalation [6] and inhibition of macromolecular biosynthesis [7]. In our research, we found that doxorubicin can down-regulate epithelial cell adhesion molecule (EpCAM) expression and decrease EpCAM-positive cell amounts in human HCC cell lines. EpCAM is an epithelium-specific, Ca^2+^ independent, cell-to-cell adhesion molecule. It is encoded by the EPCAM gene in humans and also has been designated as TACSTD1 (tumor-associated calcium signal transducer one).

EpCAM is expressed in fetal lung, kidney, liver, pancreas, skin, and germ cells, and in adult epithelia. EpCAM up-regulates the proto-oncogene c-Myc and cyclins A/E, which are involved in the cell cycle and proliferation. EpCAM over-expression is correlated with cancer malignancy and with poor survival in breast [8], ovarian [9], colon, esophageal squamous cell carcinoma [10] and squamous head and neck carcinoma cells. The function of EpCAM and its regulatory mechanism are largely unclear in HCC. Our research results showed that EpCAM is the target of doxorubicin, which can down-regulate levels of EpCAM expression and EpCAM-positive cells in HCC cell lines HepG2, Hep3B and HuH-7.

## Methods

This study involved the use of three human HCC cell lines—HepG2, Hep3B and HuH-7—which were used in accordance with the Helsinki Declaration. No human subjects were used in these studies.

### Cell culture

Hep3B and HepG2 cells were obtained from American Type Culture Collection (Rockville, MD). HuH-7 cells were purchased from Invitrogen Company (Carlsbad, CA). HepG2 and HuH-7 were grown in Dulbecco’s Modified Eagle Medium (DMEM, Invitrogen, Carlsbad, CA) supplemented with 10 % fetal bovine serum and penicillin (100 U/ml)/streptomycin sulfate (100 μg/ml) (Invitrogen, Carlsbad, CA). Hep3B was grown in Eagle’s Minimum Essential Medium (EMEM, Invitrogen, Carlsbad, CA) supplemented with 10 % fetal bovine serum and penicillin (100 U/ml)/streptomycin sulfate (100 μg/ml) (Invitrogen, Carlsbad, CA).

### MTT assay

Cell viability was measured by MTT assay. In order to reduce the influence of the chemotherapeutic reagent on MTT results, we set up blank controls for each different concentration of chemotherapeutic agents. Cells were plated on 96-well plates at a density of 1 × 10^4^ cells per well. When 90 % growing confluent reached, cells were assigned to three groups and treated with different concentrations of doxorubicin (Sigma, St. Louis, MO), 5-FU (Sigma, St. Louis, MO) and cisplatin (ALEXIS Biochemical, Lausen Switzerland) for 24, 48 h or 72 h. After treatments, 20 uL of 5 mg/mL MTT in PBS were added to each well and incubated for 4 h, and then 100 μL of lysis buffer was added. The lysis buffer consisted of 20 % SDS and 50 % dimethyl formamide [11]. The optical density (O.D.) at 570 nm was determined using a 96-well plate reader. The viability rates were calculated from the O.D. readings with various concentrations of chemotherapeutic agents using the control cells as 100 %.

### Western blotting

Same as MTT assay, cells were assigned to three groups and treated with three different chemoagents. After treatments, cells were washed twice with cold PBS and harvested on ice in lysis buffer containing 150 mM NaCl, 50 mM Tris/HCl (pH 7.6), 1 % Triton, 1 μg/ml aprotinin, and 100 μg/ml phenylmethylsulfonyl fluoride. The equivalent volume of loading buffer (100 mM Tris/HCl (pH 6.8), 4 % SDS, 20 % glycerin, 10 % β-mercaptoethanol and 0.2 % bromphenol blue) was added and mixed again. The samples were then denatured at 95 °C for 5 min. After electrophoresis, proteins were transferred to a polyvinylidene fluoride membrane. The membrane was probed with rabbit polyclonal antibodies or mouse monoclonal antibodies against Bcl-2 and caspase-3 (p34) (Santa Cruz; 1:1000 dilution), EpCAM (323/A3, Santa Cruz; 1:1000 dilution), or mouse monoclonal anti-β-Actin (Sigma 1:5000 dilution) at 4 °C overnight. After washing, the second antibody (goat anti-rabbit HRP) and donkey anti-mouse HRP (Santa Cruz; 1:2500 dilution) were added respectively. Specific antibody–antigen complexes were detected by using the ECL Western blot detection kit (Pierce). The protein bands were quantified by densitometry analysis.

### Real-Time RT-PCR (qPCR)

Cells were assigned to three groups and treated with three different chemoagents. After treatment, total RNA was extracted using the TRIzol reagent (Invitrogen). First-strand complimentary DNA (cDNA) was synthesized from total RNA according to the manufacturer’s protocol for the RNA PCR kit (Promega, Madison, WI, USA). Quantitative PCR was carried out using the ABI 7300 real-time PCR system (Applied Biosystems, Carlsbad, CA). EpCAM expression was quantified and β-actin was used as an endogenous reference. Results were expressed as fold change in gene expression.

### Flow cytometry analysis

FTIC-conjugated EpCAM monoclonal antibody (EBA-1) was purchased from Santa Cruz Company. HepG2, Hep3B and HuH-7 cells were seeded in 6-well plates, incubated at least for 24 h, and reached above 80 % confluence before chemotherapeutic agent treatment. Different concentration of doxorubicin, 5-FU and cisplatin were added to the cells and incubated for 2 days. Finally, cells were dissociated with 0.25 % trypsin-EDTA (1 mM) (Invitrogen) for 3 min and washed with fluorescence-activated cell sorting buffer (PBS containing 1 % fetal calf serum) and then incubated for 1 h at 4 °C in fluorescence-activated cell sorting buffer with the corresponding mAb: anti-EpCAM. Flow cytometry analysis was performed with a BD FACSCanto II flow cytometer (BD Biosciences).

### Xenograft mice model

Eight-weeks-old nude BALB/c mice were used for the xenograft model, and six mice were assigned to doxorubicin pretreated Hep3B group and six mice were assigned to untreated control group. Both FGF21KO and C57 BL/6 J mice were housed four per cage, given commercial chow and tap water, and maintained at 22 °C on a 12-hour light/dark cycle. To establish xenograft mice model, Hep3B cells were cultured in 75 cm^2^ flasks and pretreated with doxorubicin at 0.5 μM for 24 h. After treatment, the cells were counted, and 1 million cells were used for inoculation and 1 million cells were used for Western blot to determine the EpCAM protein levels. Doxorubicin pretreated as well as untreated Hep3B cells were inoculated at 10^6^ cells/mouse into the right flank for 4 weeks. Betadine solution swabstick will be used prior to the inoculation. Operation manipulations will be done under sterile conditions. To determine the tumor size, the length and width of tumor were measured with an accuracy of 0.01 mm using a digital caliber. Animal procedures were approved by the Institutional Animal Care and Use Committee of University of Louisville, which is certified by the American Association for Accreditation of Laboratory Animal Care.

### RNA interference

To define the link between chemotherapeutic agents and EpCAM, a small interfering RNA (siRNA) specific to TACSTD1 (SI03019667) and a negative control siRNA (1022076) were designed and synthesized by Qiagen (Qiagen, Valencia, CA). HepG2 cells were cultured for overnight at 4 × 10^5^ cells per well in a 6-well plate and 1 × 10^4^ cells per well in 96-well plates. Transfection was performed using Lipofectamine 2000 transfection reagent (Invitrogen), according to the instructions of the manufacturer. A total of 100 pmol/well of siRNA was used for 6-well plate transfection, and 5 pmol/well of siRNA for 96-well plate. After an 8-hour transfection period, it was changed into fresh medium. After 2-day incubation, cells were assigned to three groups and treated with three different concentrations of doxorubicin for another 2 days. Cells in 6-well plates were collected for Western blot analysis. Cells in 96-well plates received an additional 20 ul of MTT per well for cell viability analysis.

### Clinical rationale: chemotherapeutic choices

Doxorubicin, 5-FU, and cisplatin are agents typically chosen for the treatment of HCC. Specific concentrations of these drugs were chosen based on extrapolations from two main factors: clinically applicable dosing combined with known pharmacokinetic data (Schaaf [12], Greene [13], DeJongh [14]). The clinical dosing of these drugs is based on total body surface area, which is variable for each patient. For that reason, the dosage of these drugs was standardized based on an average patient size of 1.25 m^2^. We chose a value for each drug that was the approximate median of the range seen in multiple dosing protocols. By multiplying the dose and our standardized size, we were able to determine the number of milligrams administered to the standardized patient. This mg dosage combined with the volume of distribution available free therapeutic agent to create a theoretical chemotherapeutic concentration available in total body water. This concentration was subsequently divided by five to account for the presence of the drug in the extra-cellular fluid alone, as that would be the actual amount present in contact with tumor cells.

### Statistics

All experiments were independently performed, at least, three times to meet the assumptions of the statistical approach. The data are expressed as mean ± standard deviation (*n* = 3–6). The data were analyzed by analysis of variance (ANOVA) and Newman-Keuls’ Multiple-Comparison Test. Differences between groups were considered significant at *P* <0.05.

## Results

### Three hepatocellular carcinoma cell lines have different sensitivity to chemotherapeutic agents

**Fig. 1:**
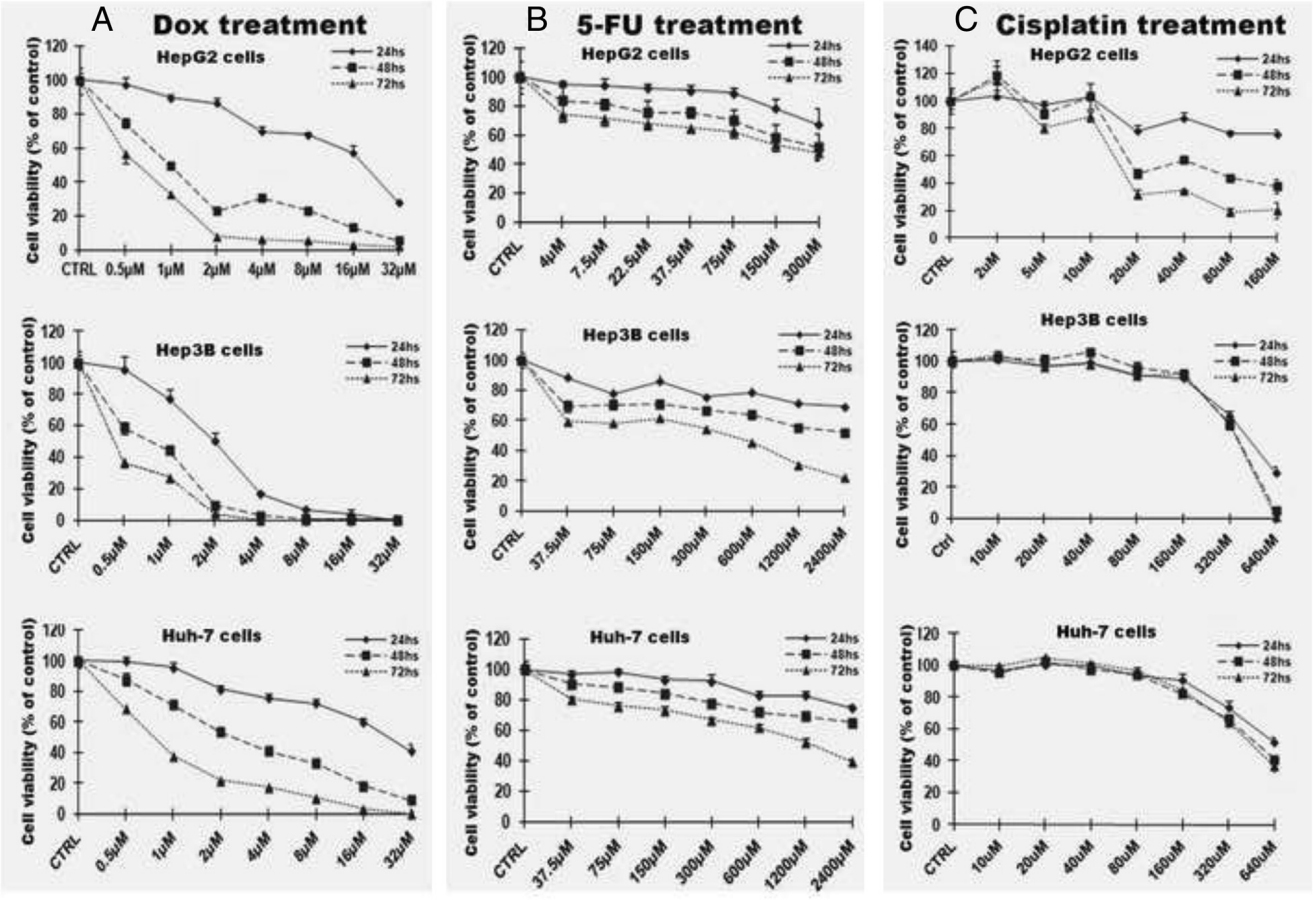
Three hepatocellular carcinoma cell lines had different sensitivity to chemotherapeutic agents. The blank controls for every different concentration of chemotherapeutic agents were set up in order to reduce the influence of the chemotherapeutic reagent on MTT results. Dox: doxorubicin; 5-FU: 5- fluorouracil

For each carcinoma cell line investigated in this study, cell viability assays were performed in order to determine their sensitivities to three chemotherapeutic agents: doxorubicin, 5-FU and cisplatin. The results indicated that all three HCC cells were sensitive to doxorubicin at lower concentrations, 0.5 and 1 μM. For 2-day exposure to 0.5 μM of doxorubicin, the cell viability of the Hep3B cell line is 58.56 %, HepG2 is 74.52 %, and HuH-7 is 87.84 %. When treated at the concentration of 4 μM doxorubicin for 3-day treatment, Hep3B were totally dead. However, HepG2 had 6.01 % of cells alive, and HuH-7 had 17.67 % of cells alive. Based on these results, the Hep3B cells are more sensitive in vitro to doxorubicin than HepG2 and HuH-7(Fig. 1a). In 5-FU treatment (Fig. 1b), the HepG2 cells show decreased viability with 5-FU treatment starting at 4 μM, but not Hep3B and HuH-7 cells. Hep3B and HuH-7 cells show decreased viability with 5-FU treatment starting at 37.5 μM. Cell viability was also determined in three HCC cell lines after exposure to cisplatin (Fig. 1c). HepG2 cells show decreased viability with cisplatin treatment starting at 10 μM. But Hep3B and HuH-7 cells show more resistant to cisplatin. Hep3B and HuH-7 cells show decreased viability with cisplatin treatment starting at 80 μM. Depending on cell-line sensitivity to the three chemotherapeutic agents, the dose is selected to treat the cells for the EpCAM expression assay.

### Doxorubicin exposure decreased EpCAM mRNA level, protein level and positive cells in HCC cell lines

**Fig. 2:**
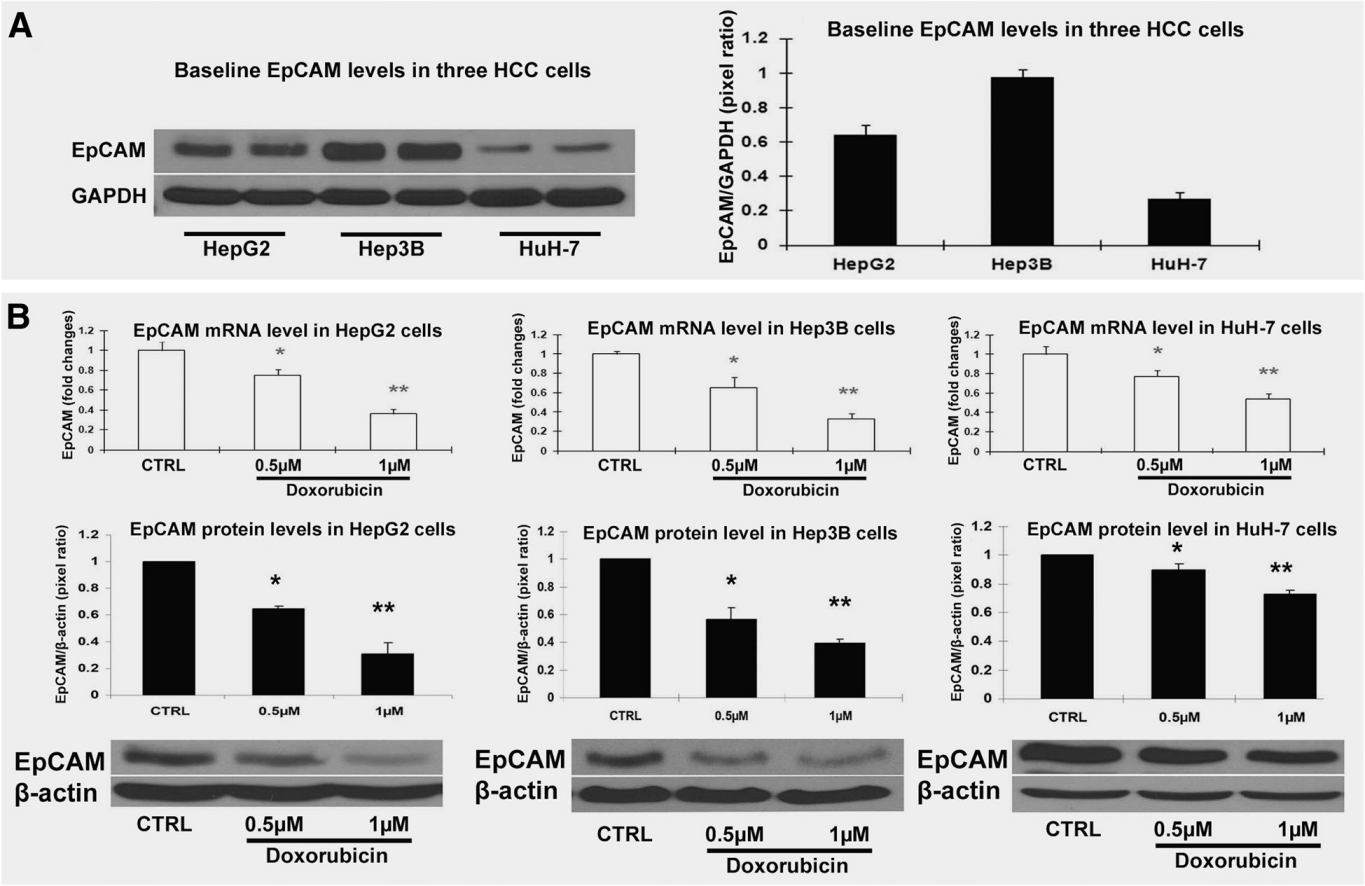
EpCAM protein expression level was decreased by doxorubicin in HCC cell lines. **a** Baseline EpCAM protein levels in HepG2 cells, Hep3B cells and HuH-7cells. The bands were scanned and analyzed with ImageQuant 5.2 software. The quantification was presented as Pixel ratio. **b** EpCAM mRNA and protein levels in HepG2 cells, Hep3B cells and HuH-7cells challenged by doxorubicin. Data are presented as mean ± SD. *p <0.05 vs control; **p <0.01 vs control

**Fig. 3:**
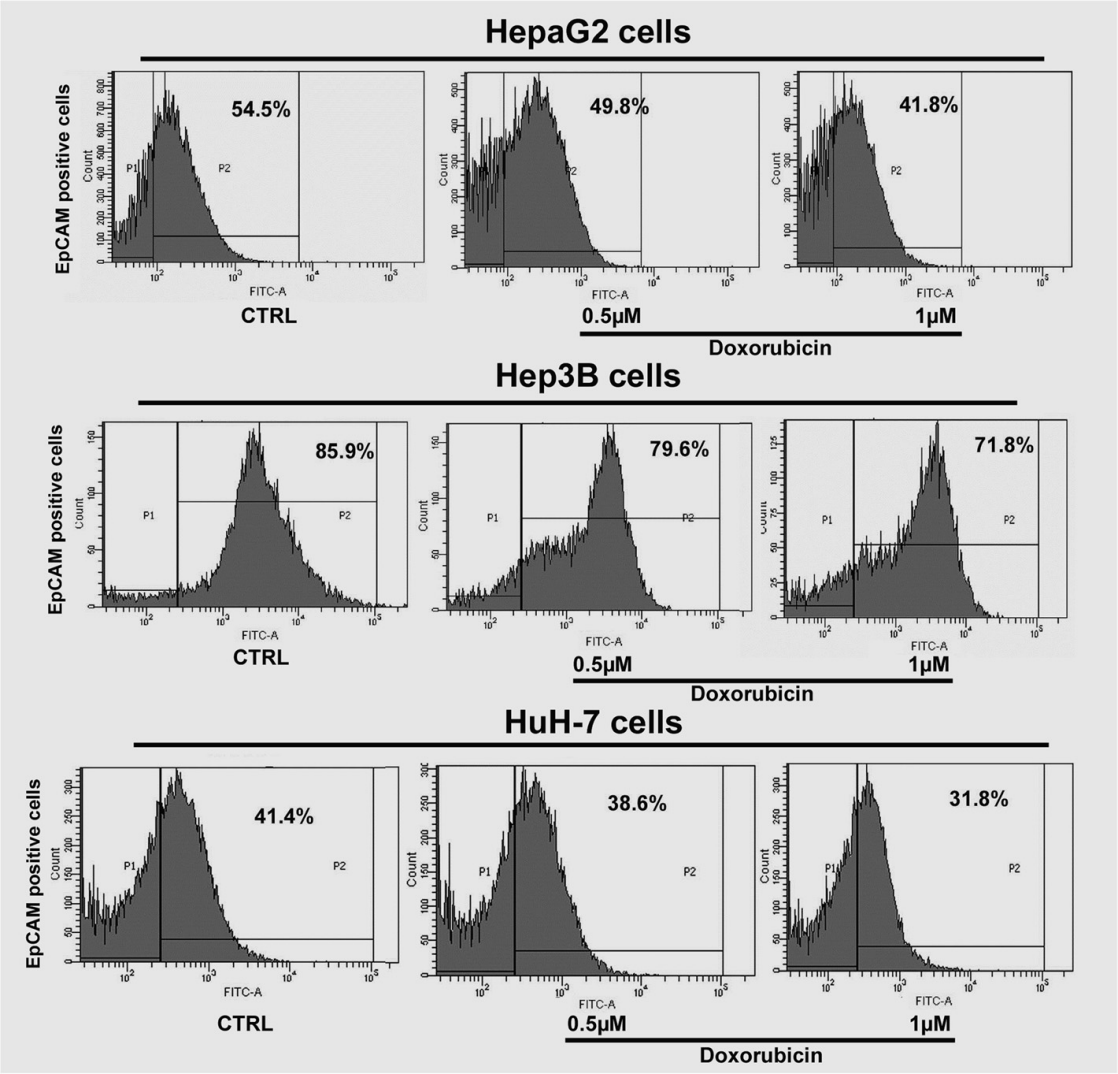
Flow cytometry analysis of EpCAM positive cells. In the baseline, Hep3B cells have a much higher percentage of EpCAM positive cells than HepG2 and HuH-7. Doxorubicin exposure decreased EpCAM positive cell percentages in HepG2, Hep3B and HuH-7 cells

First, the baseline of EpCAM expressions was evaluated at protein level. The result indicated that Hep3B cells and HepG2 cells expressed higher level of EpCAM, while the HuH-7 expressed lower level of EpCAM (Fig. 2a). When the three HCC cell lines challenged with chemotherapeutic doxorubicin at sensitive dosing of 0.5 and 1 μM which were determined previously, there were significant changes in EpCAM expression at both mRNA and protein levels. The results indicated that the EpCAM expression was significantly down-regulated by doxorubicin treatment in all three cell lines (Fig. 2b). Interestingly, the higher baseline levels of EpCAM in both Hep3B and HepG2 cells were significantly decreased by doxorubicin, and the decreases of EpCAM expressions were associated to the decreased cell viability. Flow cytometry assay was performed to further determine whether the decreased EpCAM expression was associated with decreased number of EpCAM positive cells. In the baseline, the HepG2 cells had 54.5 % of EpCAM positive cells, the Hep3B cells had 85.9 % of EpCAM positive cells, and the HuH-7 cells had 41.4 % of EpCAM positive cells (Fig. 3). This Flow cytometry result of EpCAM positive cells was consistent to the Western blot result of EpCAM protein level.

### Decreased EpCAM by doxorubicin slowed done the tumor growth in vivo

**Fig. 4:**
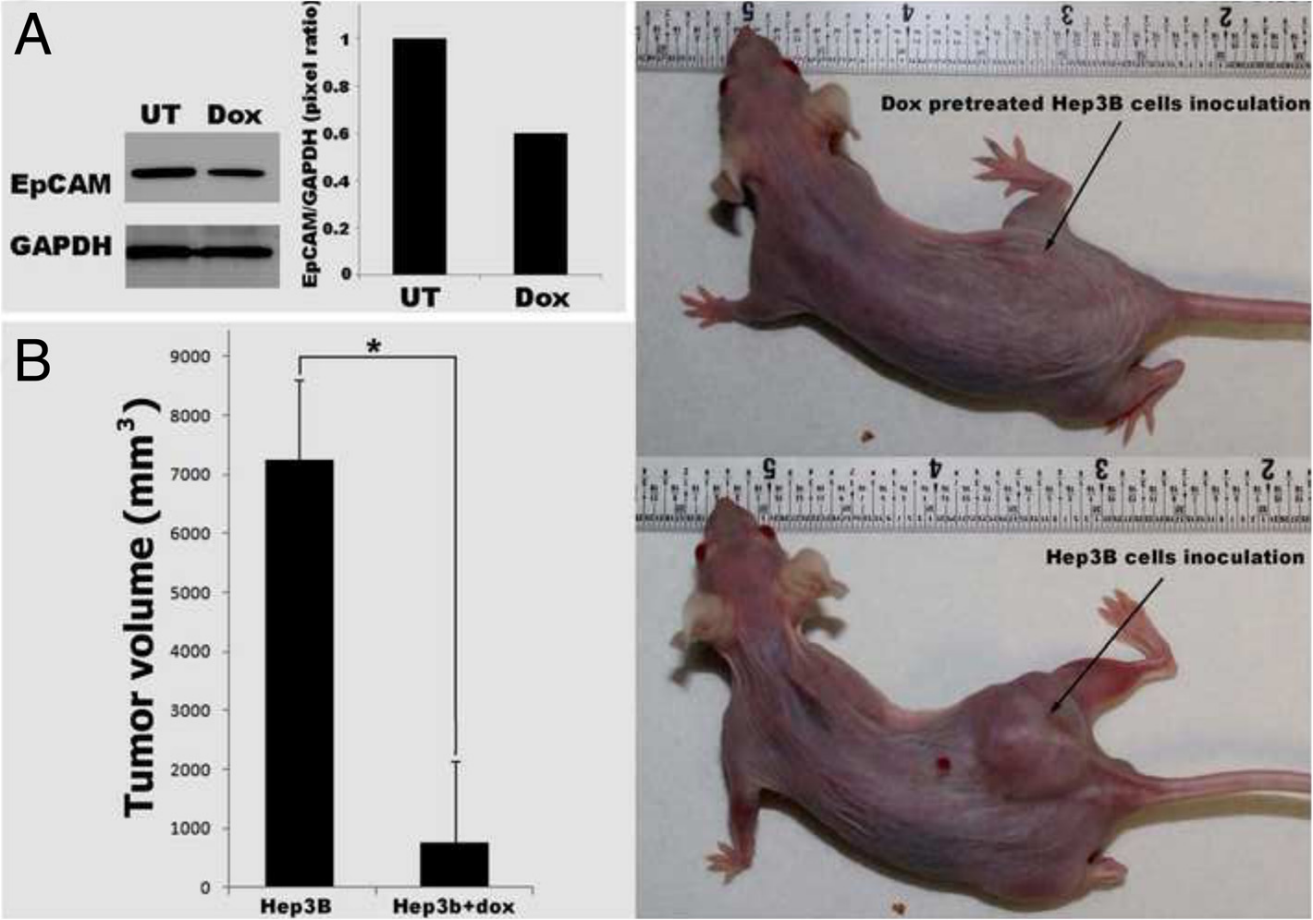
Decreased EpCAM by doxorubicin slowed done the tumor growth in vivo*.* **a** EpCAM expression was decreased in Hep3B cells after doxorubicin treatment at 0.5 μM for 24 h. **b** Doxorubicin pretreatment significantly deceased tumor size in vivo compared to untreated group. Data are presented as mean ± SD. *p <0.05 vs untreated Hep3B inoculation group

To determine whether decreased EpCAM in HCC cells would affect the tumor growth in vivo, we used Hep3B cells which were sensitive to doxorubicin for the xenograft study. The results indicated that doxorubicin pretreated Hep3B cells lost about 40 % EpCAM protein (Fig. 4a). The loss of EpCAM caused decrease of the Hep3B cell growth in vivo. As shown in Fig. 4b, the tumor sizes were significantly decreased in doxorubicin pretreated group compared to untreated group (*p* <0.05).

### Chemo-resistence is positively related to EpCAM expression in HCC cell lines

**Fig. 5:**
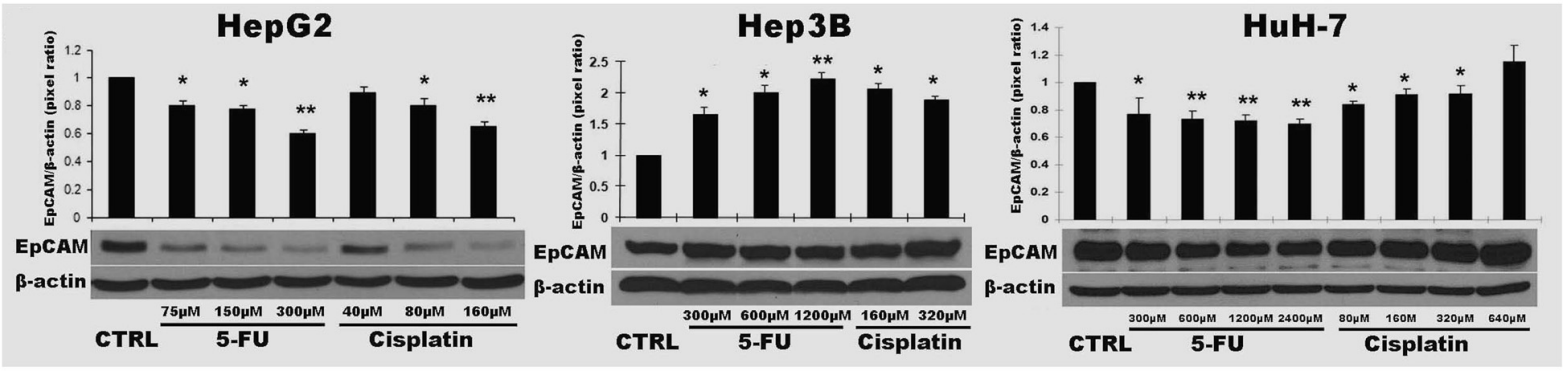
Chemo-resistence is positively related to EpCAM expression in HCC cell lines after 5-FU and cisplatin treatment. EpCAM protein levels were analyzed by Western blot. The bands were scanned and analyzed with ImageQuant 5.2 software. The optical density was modified one as control group. Data are presented as mean ± SD. *p <0.05 vs control; **p <0.01 vs control. Each experiment was repeated times times

As shown in Fig. 1, three HCC cell line, especially Hep3B and HuH-7, cells showed more resistant to 5-FU and cisplatin than doxorubicin. Therefore, we further evaluated the protein levels of EpCAM in three HCC cells challenged by 5-FU and cisplatin in Hep3B and HuH-7 cells. The result indicated that show the EpCAM protein levels of HepG2 cells decreased by both 5-FU and cisplatin at lower concentrations (5-FU: 75–300 μM; cisplatin: 40–160 μM). However, the EpCAM protein levels in Hep3B cells were increased after the challenges of 5-FU and cisplatin even at higher concentrations (5-FU: 300–1200 μM; cisplatin: 160–320 μM). Although the HuH-7 cells showed decreased EpCAM protein levels when treated with 5-FU at concentration of 300 μM, the decreases became blunt when treated at higher concentrations from 600 to 2400 μM. For the treatment of cisplatin, HuH-7 cells showed decreased EpCAM protein level when treated at concentration of 80 μM, however EpCAM protein levels were increased when treated with cisplatin at higher concentration from 160 to 640 μM (Fig. 5). Unlike doxorubicin, 5-FU and cisplatin challenged HCC cells showed a different EpCAM expression pattern, and this discrepancy implied that doxorubicin could target directly to EpCAM but not 5-FU and cisplatin.

### EpCAM knock-down attenuated cell mortality after doxorubicin exposure

**Fig. 6:**
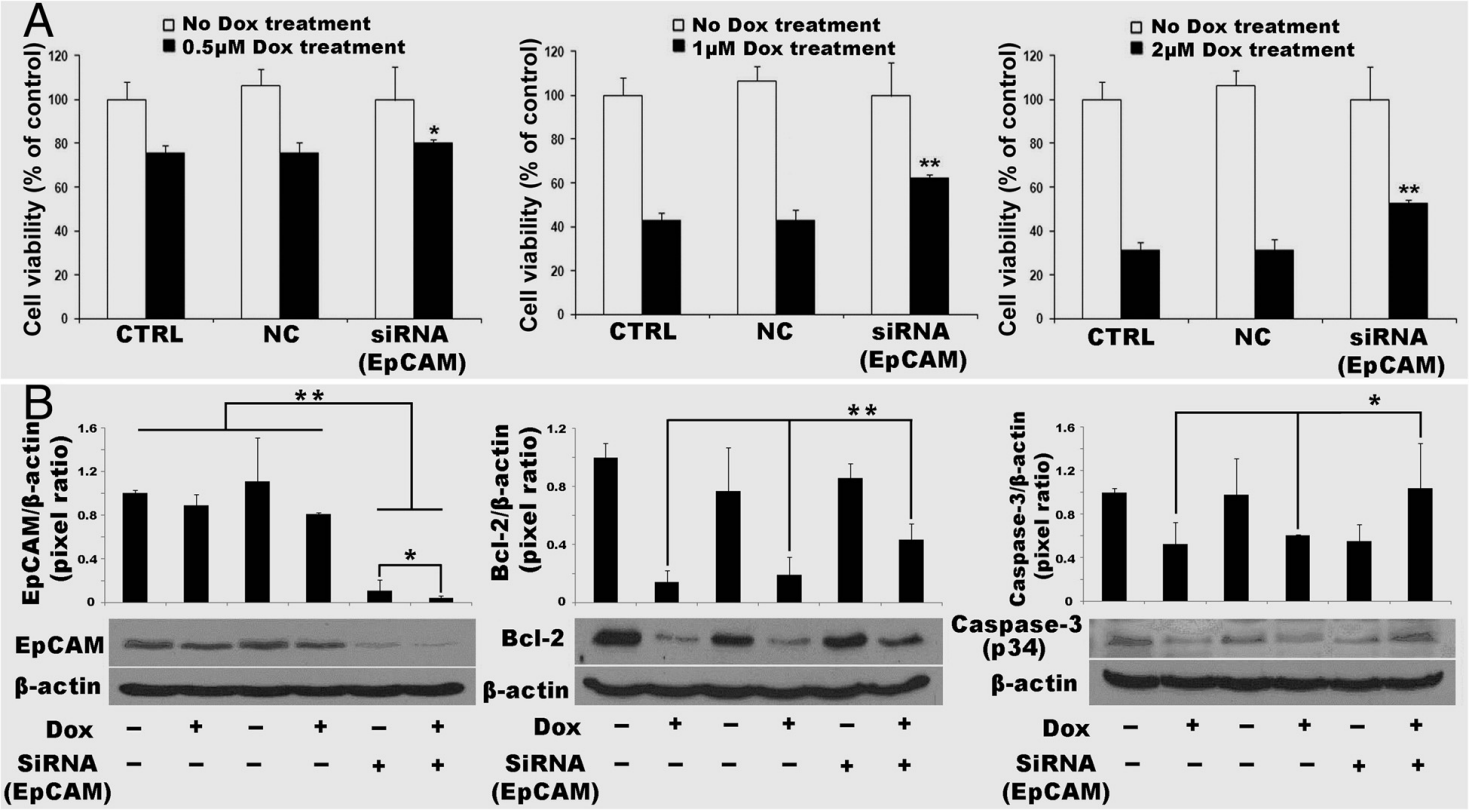
EpCAM knock-down attenuated cell mortality after doxorubicin exposure. **a** cell viability by MTT assay. **b** the protein levels of EpCAM, Bcl-2 and Caspase-3 (p34) by Western blot. *NC* negative transfection control, *Dox* doxorubicin. Data are presented as mean ± SD. *p <0.05 vs NC (Top); **p <0.01 vs NC (Top). Each experiment was repeated 3 times

To investigate if EpCAM is a target of doxorubicin, EpCAM siRNA was transfected into HepG2 cells. We selected the HepG2 cells based on the previous finding of the doxorubicin sensitivity in Fig. 1. With doxorubicin challenge, the viability of HepG2 cell (74.52 %) was either not too high or too low, in the middle between Hep3B cell (58.56 %) and HuH-7 (87.84 %). Therefore, use of HepG2 cell can avoid the experimental bias. After a 2-days incubation of EpCAM siRNA, HepG2 cells were treated with doxorubicin, at 0.5, 1 and 2 μM for an additional 2 days. In the no-doxorubicin treatment group, we modified 100 % of cell proliferation as control group. MTT assay showed the cell viabilities were significantly increased (*P* <0.05 or *P* <0.01, *vs* negative control) in all EpCAM siRNA groups after doxorubicin treatment (Fig. 6a).

As we know that apoptosis is an important mechanism for doxorubicin induced cell death, we further investigated two important effectors of cell apoptosis, Bcl-2 and Caspase-3 (p34). Bcl-2 is an anti-apoptosis protein related to cell proliferation and cell survival. Cell viability is partly recovered if Bcl-2 is increased. Caspase-3 (p34) is expressed in cells as an inactive precursor from which the p17 and p11 subunits of the mature caspase-3 are proteolytically generated during apoptosis. Western blotting results showed that EpCAM protein levels were significantly decreased in the HepG2 cells compared to that in negative transfection control and non-transfection control (*P* <0.01). When the HepG2 cells with EpCAM siRNA transfection challenged by doxorubicin, the EpCAM protein level was further decreased (*P* <0.05). Bcl-2 was decreased when the cells challenged by doxorubicin, but the Bcl-2 protein was in a higher level when EpCAM being silenced compared to that in non-transfection control and negative transfection control (*P* 0.01). Similar to Bcl-2, the inactive form of caspase-3 (p34) was decreased when the non-transfection control cells and negative transfection control cells challenged by doxorubicin. However EpCAM silenced HepG2 cells maintained a higher protein level of caspase-3 (p34), indicating that less apoptotic effectors (caspase-3 p17 and caspase-3 p11) was proteolytically generated (Fig. 6b). This showed that EpCAM knock-down made doxorubicin lose its cell-killing target.

## Discussion

In the present study, we found that doxorubicin could decrease EpCAM expression level and percentage of EpCAM positive cell population in HepG2, Hep3B and HuH-7 cell lines, and with cell viability decreasing. We used EpCAM siRNA knock-out EpCAM expression in three cell lines and found cell mortality was attenuated when cells were exposed to doxorubicin, which suggests that EpCAM was one of the targets of doxorubicin.

We first compared the viability of three cell lines, HepG2, Hep3B and HuH-7, exposed to chemotherapeutic agents. We found that in different cell lines, there are different levels of sensitivity to doxorubicin, 5-FU and cisplatin. Hep3B is more sensitive to doxorubicin than HepG2 and HuH-7. HepG2 is more sensitive to 5-FU and cisplatin than Hep3B and HuH-7. This is an insight to clinic for chemotherapy.

Epithelial Cellular Adhesion Molecule (EpCAM), also known as KS1/4, gp40, GA733-2, 17-1A, and TROP-1, is a transmembrane glycoprotein that functions as a homophilic Ca2+−independent adhesion molecule. EpCAM is a pan-epithelial differentiation carcinoma-associated antigen expressed on almost all carcinomas. EpCAM is up-regulated in the majority of human epithelial carcinomas, including colorectal [15], breast [16, 17], prostate [18], lung [19], cervical epithelium [20, 21], colon, head and neck [22], and hepatic carcinomas [23, 24]. The expression levels of EpCAM correlate with de-differentiation and malignant proliferation of epithelial cells. The level of EpCAM expression and the number of positive cells has been found to increase with the grade of carcinogenesis in cervical intraepithelial neoplasia [20]. Increasing amounts of EpCAM also has been correlated with lower life expectancy of lung cancer patients [19]. EpCAM is highly over-expressed in primary and metastatic breast cancer and associated with poor disease-free and overall survival in primary breast cancers [25].

EpCAM directly impacts cell cycle, proliferation, and metabolism and induces the protooncogene c-myc and the cell cycle regulating genes cyclinA and E [26]. Inhibition of EpCAM expression has been shown to result in a dramatic change in phenotype and a decreased proliferation of carcinoma cells [23]. Silencing of EpCAM expression decreased the migration rate [27]. EpCAM and Wnt-β-catenin act in the same signaling pathway [28].

EpCAM is used as a cancer stem cell marker [27], and as an early biomarker of hepatocellular carcinoma [29]. EpCAM is also a biomarker for hepatic stem cells [30–32]. In addition, several clinical trials targeting EpCAM have been conducted [33].

The majority of hepatocytes in 8-week embryonic liver showed EpCAM expression [24]. Abnormal liver tissue displayed a strong EpCAM expression in the epithelium of typical and atypical bile ducts. In addition, periportal or periseptal hepatocytes revealed variable staining of EpCAM, which is directly related to acute and chronic inflammatory changes. The EpCAM expression in hepatocytes was most pronounced in acute and chronic active hepatitis, with EpCAM expression levels that are common to bile ductular cells. This suggests that the hepatocytes in diseased liver represent transformed hepatocytes.

It is known that mature hepatocytes are negative for EpCAM expression. EpCAM-positive HCC displays a distinct molecular signature with features of hepatic progenitor cells. Wnt-β-catenin signaling plays a pivotal role in embryogenesis and the maintenance of stem cell growth [34] and is activated during liver development/regeneration [35, 36]. EpCAM and Wnt–β-catenin signaling are connected, and both play a role in the maintenance of hepatic cancer stem cells [37]. EpCAM is one of the direct transcriptional targets of Wnt-β-catenin signaling in normal human hepatocytes and HCC cell lines [28].

We used three human cell lines. HepG2 cells were isolated from a15-year-old Caucasian, this cell line contains the wild-type TP53 gene. There is no evidence of a hepatitis B virus genome in this cell line. Hep3B cells were isolated from an 8-year-old black juvenile. This cell line contains an integrated hepatitis B virus genome and has lost the TP53 gene. HuH-7 cells were isolated from a 57-year-old Japanese, without HBV, and partly TP53-gene mutated [38]. p53 is a tumor suppressor protein that in humans is encoded by the TP53 gene. It plays an important role in apoptosis, genetic stability, and inhibition of angiogenesis in multicellular organisms. It regulates the cell cycle and, thus, functions as a tumor suppressor that is involved in preventing cancer. It can activate DNA repair proteins when DNA has sustained damage; induce growth arrest by holding the cell cycle at the G1/S regulation point on DNA damage recognition; and initiate apoptosis if DNA damage proves to be irreparable. It is reported that wild-type p53 negatively regulates EpCAM expression [39]. In patients with chronic hepatitis B, EpCAM is up-regulated [40]. In these studies, our flow cytometry assays show that the Hep3B cell line is almost 90 % EpCAM positive expression; however HepG2 is about 50 % EpCAM positive and HuH-7 is about 45 % EpCAM positive. Since there are different baseline EpCAM positive cell levels in the three cell lines, the higher the level of positive EpCAM cells, the more sensitive to doxorubicin. Our results showed that Hep3B is more sensitive to doxorubicin than HepG2 and HuH-7. Because EpCAM is oncogene [26, 41], our in vitro data suggest that, in this way, doxorubicin is better than 5-FU and cisplatin for HCC.

## Conclusion

Chemotherapeutic agent resistance is the main obstacle to successful liver cancer treatment. Chemotherapeutic drugs kill cancer cells through apoptosis [42]. EpCAM is a biomarker of cancer stem cells, has the ability to reconstitute tumors, and is involved in tumor resistance to chemo/radiation therapy. Those characteristics help show the role of EpCAM in tumor relapse and progression. At one time, we thought EpCAM had a relationship with chemotherapeutic agent assistance [43]. In our experiment we actually found that EpCAM was up-regulated with the chemotherapeutic agent killing the cells in some cell lines. We may think that EpCAM has a role in cell survival. This is worth investigating it in the future study.

## Abbreviations

5-FU: 5- fluorouracil, a chemotherapeutic agent
dTMP: deoxythymidylate
EpCAM: epithelial cell adhesion molecule (EpCAM
HCC: hepatocellular carcinoma
Hep3B: hepatocellular carcinoma cell line
HepG2: hepatocellular carcinoma cell line
HuH-7: hepatocellular carcinoma cell line
siRNA: small interfering RNA
